# Computational models for the prediction of adverse cardiovascular drug reactions

**DOI:** 10.1186/s12967-019-1918-z

**Published:** 2019-05-22

**Authors:** Salma Jamal, Waseem Ali, Priya Nagpal, Sonam Grover, Abhinav Grover

**Affiliations:** 1 JH-Institute of Molecular Medicine, Jamia Hamdard, New Delhi, India; 2 Department of Biotechnology, Jamia Millia Islamia, New Delhi, India; 3 School of Biotechnology, Jawaharlal Nehru University, New Delhi, India

**Keywords:** Adverse drug reactions, Machine learning, Random forest, Sequential minimization optimization, Feature selection

## Abstract

**Background:**

Predicting adverse drug reactions (ADRs) has become very important owing to the huge global health burden and failure of drugs. This indicates a need for prior prediction of probable ADRs in preclinical stages which can improve drug failures and reduce the time and cost of development thus providing efficient and safer therapeutic options for patients. Though several approaches have been put forward for in silico ADR prediction, there is still room for improvement.

**Methods:**

In the present work, we have used machine learning based approach for cardiovascular (CV) ADRs prediction by integrating different features of drugs, biological (drug transporters, targets and enzymes), chemical (substructure fingerprints) and phenotypic (therapeutic indications and other identified ADRs), and their two and three level combinations. To recognize quality and important features, we used minimum redundancy maximum relevance approach while synthetic minority over-sampling technique balancing method was used to introduce a balance in the training sets.

**Results:**

This is a rigorous and comprehensive study which involved the generation of a total of 504 computational models for 36 CV ADRs using two state-of-the-art machine-learning algorithms: random forest and sequential minimization optimization. All the models had an accuracy of around 90% and the biological and chemical features models were more informative as compared to the models generated using chemical features.

**Conclusions:**

The results obtained demonstrated that the predictive models generated in the present study were highly accurate, and the phenotypic information of the drugs played the most important role in drug ADRs prediction. Furthermore, the results also showed that using the proposed method, different drugs properties can be combined to build computational predictive models which can effectively predict potential ADRs during early stages of drug development.

**Electronic supplementary material:**

The online version of this article (10.1186/s12967-019-1918-z) contains supplementary material, which is available to authorized users.

## Background

Adverse drug reactions (ADRs) are unpleasant, harmful, or unwanted effects caused by a drug and are one of the main reasons for the failure of drugs and their withdrawal from the market [1]. Although a wide range of studies are being carried out on ADRs, these remain a major health concern worldwide and pose severe challenges to public health. ADRs are the fourth primary cause of deaths in the United States resulting in 100,000 every year [2]. The traditional methods of ADR prediction are highly expensive and time consuming as the lead compounds undergo extensive testing for their safety profiles through various biochemical and cellular assays in pre-marketing stages [3]. Furthermore, during post-marketing surveillance, the data on ADRs is collected from various public databases which include reports submitted by physicians and patients’ health records which again consumes a great deal of time [4]. Thus, timely and efficient ADR prediction during initial drug discovery and development stages remains a huge problem to be addressed.

In recent years, prediction of potential ADRs has become of extreme importance and several machine learning based methods have been proposed for the prediction of potential ADRs in pre-clinical stages using the chemical features of compounds, drug targets, enzymes, transporters and pathways and information on drug side effects and therapeutic indications. Azuaje et al. [5, 6] generated drug-target interaction networks for the prediction of cardiovascular ADRs of non-cardiovascular drugs. One approach proposed by Pauwels et al. [7] involved the use of chemical structures of drugs for the generation of machine-learning models using four algorithms (nearest neighbors, support vector machines (SVMs), canonical correlation analysis, ordinary, and sparse) for ADR prediction tasks and also extracted associated sets of chemical fragments and side effects. Kuang et al. [8] compared and analyzed the existing methods for drug ADRs prediction and proposed a new algorithm, the general weighted profile method, from already existing algorithms by combining their formulas and converting them into a linear model for prediction of ADRs. Cami et al. [9] constructed a bipartite network that represented drugs, side effects and their associations and then predicted adverse drug events using pharmacological network models relying on a logistic regression algorithm. In another study, Liu et al. [10] carried out large scale prediction of ADRs integrating several features of drugs that included drug targets and pathways, chemical properties of drugs, therapeutic indications, and data from other known ADRs. Huang et al. [11] generated SVMs and logistic regression prediction models trained on the combination of drug targets, gene ontology annotations, and protein–protein interaction networks. Zhang et al. [12] considered ADR prediction as a multi-label learning task and proposed a novel approach, ‘feature selection-based multi-label k-nearest neighbor method’ that led to simultaneous predictions of relevant features, as well as the generation of highly accurate prediction models.

Although numerous computational methods have been projected so far for ADR prediction problems, a lot can still be improved. The data used for generating the models is severely imbalanced, and imbalanced data classification would lead to predictions biased towards the negative class which is typically the dominating class. Another problem is with the large number of features associated with the drugs: some may be redundant, and not all would be related to ADRs. Recently, feature selection techniques have been increasingly used to identify significantly contributing features from high dimensional feature data sets for improving upon the prediction performances [13–15]. The benefits of feature selection for learning can include a reduction in the amount of data needed to achieve learning, improved predictive accuracy, learned knowledge that is more compact and easily understood, and reduced execution time. Also, we have, in our previous studies, already shown how feature selection based models have either outperformed the models generated using all the features or have given very similar results [16–20]_ENREF_15. Earlier, we used the machine-learning based methods to predict neurological ADRs by integrating the chemical, biological, and phenotypic features of drugs [21]. In the present study, without using feature selection the dimension of the files was too large to handle as is evident from initial number of features listed in Table 1, and consumed large computational power and time to generate the models. Table 2 provides a comparison of the number of drugs and types of features used in the present study and for other ADR prediction studies. It has been reported in various studies that the imbalance in the input datasets could result in biased predictions as most of the classifier are biased towards the major classes and hence show very poor classification rates on minor classes. It is also possible that classifier predicts everything as major class and ignores the minor class [22, 23]. Thus we used SMOTE technique to overcome the imbalanced data problem and ensure unbiased classification.

**Table 1:** Provides the types of features used to generate the models and the number of features obtained after RemoveUseless and mRMR selection approaches

Type of feature	Source	Initial number	RemoveUseless	mRMR	Total final features
Biological					
Targets	DrugBank	1264	1207	50	150
Transporters	DrugBank	86	84	50	
Enzymes	DrugBank	182	182	50	
Chemical					
Substructures	PubChem	881	629	50	50
Phenotypic					
Other ADRs	SIDER	5497	5292	50	100
Therapeutic indications	SIDER	1840	1600	50

**Table 2:** Provides a comparison of the number of drugs and types of features used in the present study and for other ADR prediction studies

Dataset	Drugs	Side effects	Substructures	Targets	Transporters	Enzymes	Pathways	Indications
Pauwels et al. 2011	888	1385	881	NA	NA	NA	NA	NA
Wang et al. 2014	799	1385	881	775	NA	NA	NA	719
Zhang et al. 2015	569	4192	NA	NA	NA	NA	NA	NA
Kuang et al. 2014	404	461	NA	NA	NA	NA	NA	NA
Huang et al. 2011	578	1447	NA	3880	NA	NA	NA	NA
Zhang et al. 2015	1080	2260	881	1046	96	160	268	2537
Liu et al. 2013	832	1384	881	786	72	111	173	869
Present study	965	5497	881	1264	86	182	NA	1840

**Fig. 1:**
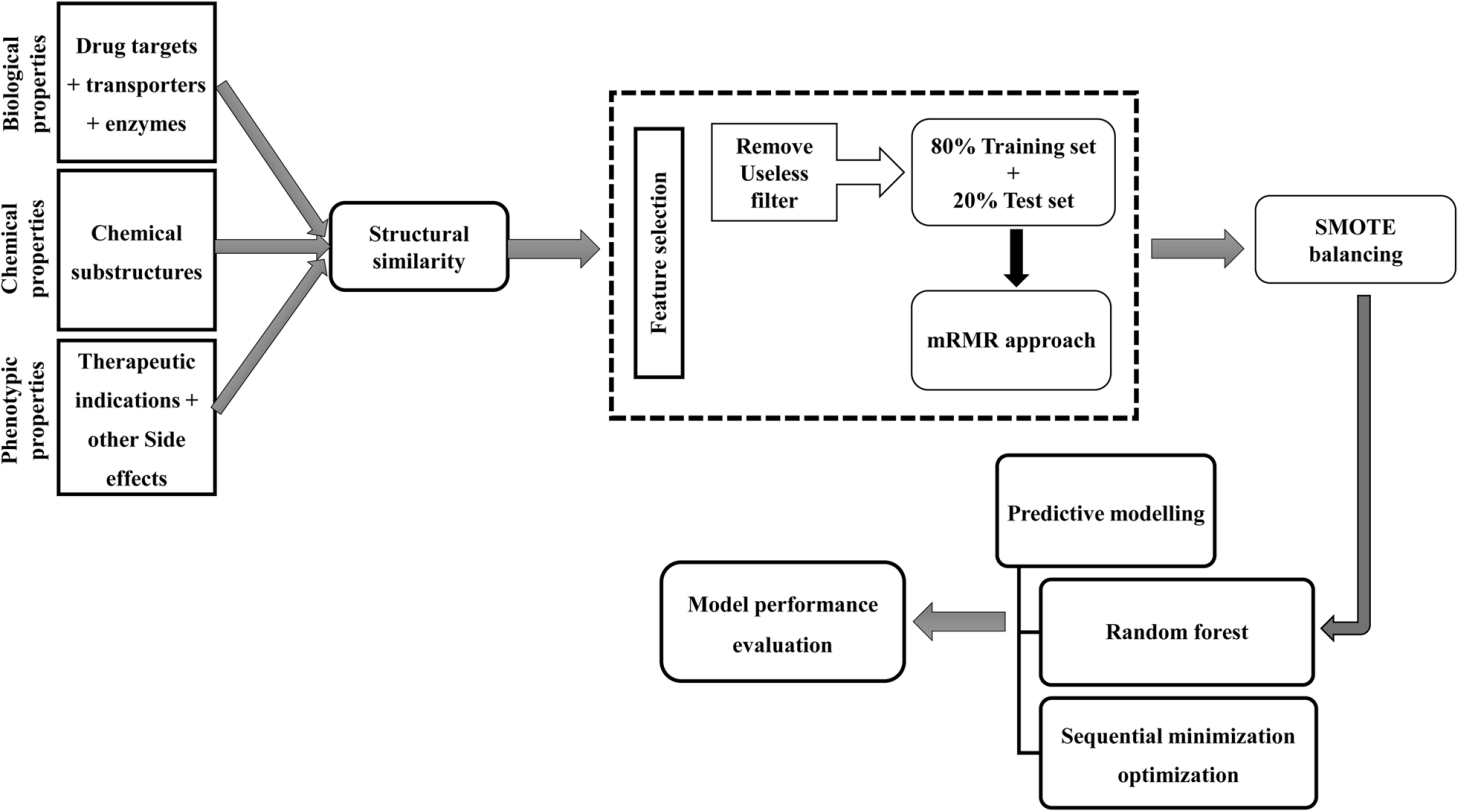
Depicts the outline of the computational methodology followed in the present study

In the present study, we have tried to solve the problem of data imbalance using the synthetic minority over-sampling technique (SMOTE) balancing method [24], and under-sampling using SpreadSubsample method. We also used a minimum redundancy maximum relevance (mRMR) [25] approach for identifying important and non-redundant features. The outline of the computational workflow followed in this paper has been shown in Fig. 1.

## Materials and methods

### Data sets

The dataset of approved drugs was retrieved from the DrugBank [26] database. DrugBank is a publicly available resource containing complete information on drugs and their actions, targets and structures. The data structural information for drugs for a total of 2141 drugs was obtained in structural data format.

### Side-effects

SIDER [27] (SE resource) is a freely accessible database containing information on marketed drugs and their documented adverse effects. As reported on October 2015, the SIDER database, version 4.1, contained data on 1430 drugs and 5880 side effects, with a total of 139,756 drug-SE associations, where each drug had an average of 39% side effects. In the present work, the complete SIDER database was obtained from http://sideeffects.embl.de/. The 2141 approved DrugBank drugs were mapped to SIDER using PubChem compound IDs (CID) as SIDER uses STITCH compounds IDs which were converted to PubChem CIDs according to the rule mentioned in (ftp://xi.embl.de/SIDER/2015-10-21/, Accessed April 2, 2018). The SIDER database was also used to collect data on therapeutic indications of drugs. We obtained information for a total of 970 drugs, which comprised data for 5497 side effects and 1840 therapeutic indications.

### Biological features from DrugBank

The biological information of drugs was comprised of drug targets, transporters (for drug transportation) and enzymes (for drug metabolism). We obtained data for 1264 drug targets, 86 transporters, and 182 enzymes, for a total of 970 drugs directly from DrugBank database.

### Chemical structure fingerprints

PaDEL [28] software was used to generate 881 PubChem [29] structure fingerprints for each of the 970 drugs in order to obtain chemical information. A substructure is part of a chemical structure, and a fingerprint is a well-ordered list of binary (0/1) bits; these bits are the Boolean representations for the presence of element counts, ring systems, atom pairs, etc. in the chemical structures.

### Chemical structural similarity between drugs

Tanimoto coefficient (TC) was calculated to measure the similarity between two drug molecules using the ChemmineR [30] platform of R interface. The chemical structures of drugs in structural data format (SDF) were used to compute atom pair descriptors for all of the drug compounds. Further similarity search was performed using the cmp.search function, with a cut-off value of 0.3, which searched the entire atom pair database generated in the previous step for structurally similar compounds. The compounds having similar chemical structures were removed from the dataset.

### Features construction

In classification models generation, one of the most important steps is to generate features which are a quantifiable property of an instance being classified. In case where the instance is a molecule, the chemical information of the molecule is converted to molecular descriptors, which are the mathematical depictions of chemical compounds. In the present study, we have used six types of features which include the molecular 2D fingerprints denoting chemical features, biological targets, transporters, enzymes associated with drugs representing biological information, therapeutic indications, and other known side effects comprising phenotypic properties of drugs. Each drug, associated with three types of properties, chemical, biological and phenotypic, was represented as a binary matrix, the elements of which were either 1 or 0, respectively indicating the presence or absence of each feature: drug targets, transporters, enzymes, 881 PubChem substructures, remaining known ADRs, and therapeutic indication. To this end, we had a 5497 × 1840 dimensional binary matrix representing phenotypic properties, a 1264 × 86 × 182 dimensional binary matrix demonstrating biological features, and an 881 dimensional binary matrix denoting chemical features for each of the total 965 drugs.

### Feature selection

A high dimensional feature space is generated for a small size of samples by the feature construction method. This may result in over fitting of the classification models while contributing to increase in dimensionality of the dataset consequently leading to increased computational time involved. Thus in the present study, a *RemoveUseless* filter available from Weka [31], a machine learning platform, was used to remove the features (chemical, biological, and phenotypic) having uniform values for all the drug molecules throughout the data set.

### Minimum redundancy maximum relevance approach

As mentioned above, a large feature space was generated where each drug was represented by large numbers of features; however not all the features contribute significantly towards classification. Choosing independent, informative, and discriminative features is very important for classification purposes; thus in the present study we used an mRMR approach as the feature selection technique to extract useful features. The mRMR approach selects features having a high correlation with the output class, while with low inter-correlation. F-statistic is used to calculate the correlation with the output class (relevance), and the Pearson correlation coefficient is used to compute correlation amongst the features (redundancy). Two lists of features are generated by this approach: the MaxRel list and mRMR features list, where MaxRel gives the significantly contributing features and the mRMR list contains the features having higher ranks for contribution with least redundancy. An mRMR approach was used with default (fifty features) and eighty features of each kind were selected for model building in the present study.

### Experimental setup

The ADRs prediction problem was considered as a binary classification problem where each drug was either associated with a particular SE (Yes) or not (No). Thus a column titled *Outcome* was appended in all the comma separated value (csv) files for the chemical, biological, and phenotypic features. Before the model generation task, the already existing 124 CV drugs were removed from the dataset and kept as a control to evaluate the performance of the generated classifier models. Machine learning is based on adapting from previous experiences and known data properties and then making predictions on the new unseen data. The feature data set was split into 80% training data (used for generation of classifier models) and 20% testing set (used for model performance evaluation) using in-house Perl script. The mRMR based feature selection was performed on training data and the testing dataset was used as a complete held out data to eliminate any biasness in classification. For each of the 36 cardiovascular ADRs, a classifier model was generated using training data that included features for the resulting 842 drugs. The predictive computational models were generated for each of the individual set of features (chemical, biological, and phenotypic) and their combinations that involved biological + chemical, biological + phenotypic, chemical + phenotypic and biological + chemical + phenotypic feature combinations.

### Handling the imbalance amongst output class

The dataset was highly imbalanced in the present study where the instances were dominated by the negative class which could lead to overestimated performance predictions biased towards the negative (dominating) class. In the present study, we incorporated SMOTE (Synthetic Minority Over-sampling Technique), under sampling of the majority class and cross validation balancing methods to alleviate the problem of data imbalance.

### SMOTE

To improve upon the performance of the classifier models, we employed a balancing strategy, SMOTE method from Weka, to bring a balance between the majority and minority classes. The SMOTE method oversamples the minority class by generating synthetic examples using the information available from the data set and resampling the data. To oversample, a sample from the minority class in the dataset is taken along with its k-nearest neighbors using Euclidean distance. Next, the difference is computed between the input vector and its nearest neighbor, which is multiplied to a random number that lies between 0 and 1 and further added to the current data point under consideration.

### Under-sampling using SpreadSubsample

In the present work, we used SpreadSubsample filter of Weka to under-sample the dominating class to introduce balance between the majority and minority class. The SpreadSubsample filter method produces a random subsample of the dataset and specifies the maximum distribution between the minority and majority class.

### Tenfold cross validation

We used tenfold cross validation in the present work to evaluate the performance of the generated predictive models. In tenfold cross validation, the original dataset was randomly partitioned into 10 equivalent sized folds or subsamples. One of the subsamples was retained as the validation set and the left overs were used as part of the training set. The process was repeated ten times (tenfold) until each subsample had been once used as validation data. At the end, the results obtained from all tenfold were averaged to get a single result.

### Machine learning models construction

Two different machine-learning algorithms were used for predictive modelling in the present study: random forest (RF) and sequential minimization optimization (SMO) algorithm. Predictive models were implemented using Weka, which is a machine learning platform that supports training models using several machine learning algorithms and their evaluation.

### Random forest

RF is an ensemble classifier developed by Leo Breiman that involves integration of multiple decision trees. A multitude of trees is generated during training of the learning model and the output class is the mode of the classes predicted by the individual trees. The larger the number of trees, the higher the accuracy. For prediction of a test case, each tree estimates an outcome which is stored for voting, and the highest-voted prediction is the final output class for the test case.

### Sequential minimization optimization

SMO algorithm, invented by John Platt, is an implementation of support vector machines (SVM) in Weka. The SMO algorithm solves the problem of quadratic programming (QP) by breaking the large QP problem into a sequence of smaller QP problems, further solving the smallest optimization problem at each step. SVMs are supervised learning models which perform binary linear classification by constructing a hyperplane, or a set of hyperplanes in high dimensional space, and try to categorize the examples to either of the two classes. The two classes are separated by the maximum possible gap; new test cases are then mapped to the same space, and a prediction is made based on the category in which the test instance falls.

## Model performance assessment

For each CV SE, classifier models were generated using RF and SMO algorithms and were evaluated using tenfold cross validation on 842 drugs. The performance of the predictive models was assessed using accuracy (ACC), precision, recall, F-measure, and Area under Curve (AUC) value. AUC value is a single measurement obtained from the receiver operating characteristic (ROC) curve, which is a plot of sensitivity or true positive rate (TPR) against false positive rate (FPR) or (1-specificity).

1 $${\text{ACC }} = \frac{\text{TP + TN}}{\text{TP + TN + FP + FN}}$$

2 $${\text{Precision }} = \frac{\text{TP}}{\text{TP + FP}}$$

3 $${\text{Recall }} = \frac{\text{TP}}{\text{TP + FN}}$$

4 $${\text{F}}_{ 1} = \frac{{ 2 * {\text{Precision*Recall}}}}{\text{Recall + Precision}}$$

where TP is true positive, FP is false positive, TN is true negative and FN is false negative.

## Results

In the present study, a total of 504 machine-learning based classifier models were generated for 36 CV ADRs using chemical, biological, and phenotypic features and their two and three level combinations for 842 drugs. Table 3 lists the 36 CV ADRs and their SIDER IDs for which the RF and SMO models were generated.

**Table 3:** Lists the 36 cardiovascular ADRs along with their SIDER ids for which the RF and SMO models were generated

Cardiovascular side effect	SIDER id
Arrhythmia	C0003811
Atrioventricular block	C0004245
Atrioventricular block complete	C0151517
Atrioventricular block first degree	C0085614
Atrioventricular block second degree	C0264906
Block heart	C0018794
Bradycardia	C0428977
Cardiac arrest	C0018790
Cardiac death	C0376297
Cardiac disorder	C0018799
Cardiac failure acute	C0264714
Cardiac failure congestive	C0018802
Cardiac failure	C0018801
Cardiac fibrillation	C0232197
Cardiac flutter	C0016385
Cardiac murmur	C0018808
Cardiac output decreased	C0007166
Cardiac tamponade	C0007177
Cardiac valve disease	C0018824
Cardiogenic shock	C0036980
Cardiomegaly	C0018800
Cardiomyopathy	C0878544
Cardiopulmonary failure	C1444565
Cardio-respiratory arrest	C0600228
Cardiotoxicity	C0876994
Cardiovascular disorder	C0007222
Conduction disorder	C0264886
Cor pulmonale	C0034072
Decompensation cardiac	C1961112
Heart malformation	C0018798
Heart rate irregular	C0237314
Heartburn	C0018834
Left ventricular failure	C0023212
Myocardial ischaemia	C0151744
Shock	C0036974
Tachycardia	C0039231

### Analysis of the features used to study CV ADRs

As described in the methods section, chemical, biological and phenotypic features of dimensions 1,532, 881, and 7337, respectively, were used to represent 842 drugs which resulted in a very high dimensional feature space. This increased the probability of redundant and irrelevant feature vectors among the feature sets. Thus we used the *RemoveUseless* filter and mRMR approach, with default parameters, to obtain non-redundant and significant features. In the case of the mRMR approach, the features with scores larger than zero were selected for generating the trained classifier models. The top 50 features, which is the default number of features for mRMR, from each of the six types—enzymes, targets, transporters, substructure fingerprints, indications and side effects—investigated in the present study were used to generate the machine learning models. Table 1 provides the types of features used for the generation of the machine learning models and the number of features obtained after *RemoveUseless* and mRMR selection approaches. The list of the top 50 features ranked by mRMR has been provided as Additional file 1.

### Performance comparison of different machine learning algorithms

For the ADRs prediction task, the following feature combinations were used as input: (1) biological features (protein targets, transporters and enzymes, 150 dimensional); (2) chemical structures (50 dimensional); (3) phenotypic properties (therapeutic indications and other known ADRs, 100 dimensional); (4) biological + chemical features (200 dimensional); (5) biological + phenotypic features (250 dimensional); (6) chemical + phenotypic features (150 dimensional); and (7) biological + chemical + phenotypic features (300 dimensional). Two different machine learning algorithms, RF and SMO, were employed for the generation of models using training data sets which were evaluated using tenfold cross validation. Table 4 provides the overall tenfold cross-validation performance of the models generated using training dataset with biological, chemical, and phenotypic features and the combination of the two and three levels of features. It is clearly evident from Table 4 that most of the AUC values are not around 0.50 which indicates that the models generated in the present study are not random predictors. Additional file 2 provide the tenfold cross-validation performance measures for the RF and SVM models for each cardiovascular ADR using biological, chemical, phenotypic features and their two and three level combinations.

**Table 4:** Provides the overall tenfold cross-validation performance of the models generated using training dataset with biological, chemical, and phenotypic features and the combination of the two and three levels of features

Type of feature	RF						SMO					
	ACC	Precision	Recall	F-score	AUC	PRC	ACC	Precision	Recall	F-score	AUC	PRC
Biological	78.11	0.77	0.99	0.87	0.62	0.81	76.73	0.76	0.99	0.86	0.58	0.73
Chemical	83.34	0.84	0.97	0.89	0.78	0.89	76.32	0.78	0.94	0.85	0.68	0.74
Phenotypic	77.06	0.75	1.00	0.86	0.54	0.75	77.91	0.77	1.00	0.87	0.54	0.74
Biological + chemical	84.80	0.86	0.95	0.90	0.81	0.91	78.87	0.80	0.95	0.86	0.72	0.75
Biological + phenotypic	80.87	0.80	0.99	0.88	0.66	0.82	79.13	0.78	0.99	0.87	0.63	0.75
Chemical + phenotypic	83.69	0.84	0.97	0.89	0.79	0.90	77.79	0.79	0.96	0.86	0.69	0.75
Biological + chemical + phenotypic	85.25	0.85	0.96	0.90	0.82	0.91	89.07	0.94	0.95	0.94	0.47	0.75

Tables 5 and 6 provide the overall performance measures for the classifier models generated using chemical, biological, and phenotypic features and the combination of the two and three levels of features using SMOTE and SpreadSubsample method. In few cases where the AUC score is around 0.5, the probable reason may be due to the fact that only a few drugs in the dataset have these side-effects which makes the prediction difficult. The training set was balanced for minority class using SMOTE and SpreadSubsample method which resulted in significant AUC values in case of cross validated models. And thus most of the AUC values in case of testing data are around 0.50 owing to the low frequency of side effects. It is evident from Table 5 that both RF and SMO models had high performance metrics; however, RF performed better that SMO in the case of chemical feature models, as well as in cases where chemical features were combined with other features. The chemical feature SMO models had an accuracy of 88.75% which was much less as compared to chemical feature RF models where the accuracy was 91.41% in SMOTE models. The accuracy of chemical feature models generated using under-sampling method was 93.69% and 93.32% in case of SMO and RF models respectively. Similar was the accuracy value in case of biological + chemical (SMO 89.06%, RF 90.24%), chemical + phenotypic (SMO 90.54%, RF 91.49%) and biological + chemical + phenotypic (SMO 89.07%, RF 90.92%) combinations models using SMOTE method.

**Table 5:** Provides the overall performance measures for the models generated using biological, chemical, and phenotypic features and the combination of the two and three levels of features on non-redundant testing dataset using over sampling of minority class

Type of feature	RF						SMO					
	ACC	Precision	Recall	F-score	AUC	PRC	ACC	Precision	Recall	F-score	AUC	PRC
Biological	93.56	0.94	0.99	0.96	0.52	0.93	91.24	0.93	0.99	0.96	0.51	0.93
Chemical	91.41	0.94	0.97	0.95	0.52	0.94	88.75	0.94	0.95	0.93	0.48	0.93
Phenotypic	93.83	0.95	1.00	0.97	0.50	0.93	93.83	0.94	1.00	0.97	0.50	0.93
Biological + chemical	90.24	0.94	0.96	0.94	0.53	0.94	89.06	0.94	0.95	0.94	0.53	0.93
Biological + phenotypic	93.66	0.94	1.00	0.97	0.52	0.94	93.30	0.94	0.99	0.96	0.52	0.93
Chemical + phenotypic	91.49	0.94	0.97	0.95	0.50	0.94	90.54	0.94	0.96	0.95	0.48	0.93
Biological + chemical + phenotypic	90.92	0.94	0.96	0.95	0.54	0.95	89.07	0.94	0.95	0.94	0.47	0.93

**Table 6:** Provides the overall performance measures for the models generated using biological, chemical, and phenotypic features and the combination of the two and three levels of features on non-redundant testing dataset using under sampling of majority class

Type of feature	RF						SMO					
	ACC	Precision	Recall	F-score	AUC	PRC	ACC	Precision	Recall	F-score	AUC	PRC
Biological	93.90	0.88	0.91	0.89	0.46	0.94	92.15	0.88	0.91	0.89	0.45	0.94
Chemical	93.32	0.85	0.88	0.87	0.48	0.94	93.69	0.85	0.89	0.87	0.44	0.93
Phenotypic	93.65	0.85	0.88	0.87	0.44	0.93	93.85	0.85	0.89	0.87	0.44	0.93
Biological + chemical	93.07	0.85	0.88	0.86	0.46	0.94	93.72	0.85	0.89	0.87	0.44	0.93
Biological + phenotypic	93.51	0.85	0.88	0.87	0.43	0.93	93.82	0.85	0.89	0.87	0.44	0.93
Chemical + phenotypic	93.43	0.85	0.88	0.87	0.45	0.94	93.83	0.85	0.89	0.87	0.44	0.93
Biological + chemical + phenotypic	93.06	0.85	0.88	0.86	0.47	0.94	93.61	0.85	0.89	0.87	0.44	0.93

As mentioned above, different features were combined to evaluate their predictive capacities for CV ADRs prediction task. The chemical features models were least informative in the case of both RF and SMO classification models generated using SMOTE method. The biological and phenotypic models were much more accurate than chemical features models with accuracy values of 93.56%, 93.83% and 91.41% in case of RF and 93.28%, 88.75% and 93.83% for SMO models respectively. The accuracy of the models showed significant improvement upon addition of phenotypic features from 88.75 to 90.54% in case of SMO models. The biological and phenotypic combination models yielded better accuracy values (93.66% RF and 93.30% SMO). However the phenotypic features were most accurate and informative, leading to highly predictive models indicating that phenotypic features played an important role in the ADR prediction task. On the other hand the RF and SMO models generated using biological, chemical, and phenotypic and combination models using SpreadSubsample under-sampling method had almost similar performance. Additional files 3 and 4 provide the performance measures for the RF and SVM models for each cardiovascular ADR using fifty biological, chemical, phenotypic features and their two and three level combinations on non-redundant testing dataset using SMOTE and SpreadSubsample data balancing method respectively.

In addition we also generated biological, chemical, phenotypic and combination models using eighty features. The models generated using eighty features performed largely better in terms of accuracy in comparison to the models generated using the default fifty features (Table 7). All the models were around 93–94% accurate and had precision, recall, F-measure and PRC values in the range of 93–100%. However the RF biological and phenotypic combination models performed best in terms of AUC value (0.72). The individual performance metrics for RF and SMO generated on non-redundant testing dataset using eighty mRMR biological, chemical, phenotypic features and their combinations for 36 CV ADRs has been provided as Additional file 5. Additionally we have also compared the statistical performances of models generated in the present study with other approaches for drug ADR prediction (Table 8).

**Table 7:** Provides the overall performance measures for the models generated using eighty biological, chemical, and phenotypic features and the combination of the two and three levels of features on non-redundant testing dataset

Type of feature	RF						SMO					
	ACC	Precision	Recall	F-score	AUC	PRC	ACC	Precision	Recall	F-score	AUC	PRC
Biological	93.76	0.94	0.99	0.96	0.48	0.94	93.59	0.93	0.99	0.96	0.50	0.93
Chemical	93.03	0.93	0.98	0.95	0.54	0.95	93.98	0.93	100.00	0.96	0.50	0.93
Phenotypic	94.85	0.95	0.98	0.97	0.69	0.96	94.23	0.94	0.99	0.96	0.52	0.94
Biological + chemical	93.81	0.94	0.98	0.96	0.55	0.95	93.94	0.93	0.99	0.96	0.50	0.93
Biological + phenotypic	94.31	0.94	0.99	0.96	0.72	0.96	94.57	0.94	0.99	0.97	0.50	0.94
Chemical + phenotypic	94.97	0.95	0.99	0.97	0.72	0.97	94.16	0.94	0.99	0.95	0.52	0.94
Biological + chemical + phenotypic	94.28	0.94	0.99	0.96	0.68	0.96	92.34	0.95	0.99	0.97	0.51	0.95

**Table 8:** Provides the comparison of performances of models generated in the present study with other approaches for drug ADR prediction

Dataset	Feature	Algorithm	AUC	Sensitivity/recall	Precision	Accuracy
Pauwels et al. 2011	Substructures	RF	0.62	0.97	0.93	91.30
		SMO	0.50	1.00	0.92	92.42
Present study	Biological	Random forest	0.52	0.99	0.94	91.24
	Chemical		0.52	0.97	0.94	88.75
	Phenotypic		0.5	1.00	0.95	93.83
	Biological + chemical		0.53	0.96	0.94	89.06
	Biological + phenotypic		0.52	1.00	0.94	93.3
	Chemical + phenotypic		0.5	0.97	0.94	90.54
	Biological + chemical + phenotypic		0.54	0.96	0.94	89.07
	Biological	Support vector machine	0.51	0.99	0.93	93.56
	Chemical		0.48	0.95	0.94	91.41
	Phenotypic		0.5	1.00	0.94	93.83
	Biological + chemical		0.53	0.95	0.94	90.24
	Biological + phenotypic		0.52	0.99	0.94	93.66
	Chemical + phenotypic		0.48	0.96	0.94	91.49
	Biological + chemical + phenotypic		0.47	0.95	0.94	90.92

### Case study on cardiovascular drugs

To exhibit the realistic application and clinical importance of the generated predictive models, the models were evaluated for their ability to predict the already reported ADRs of the known CV drugs. Already known CV drugs were removed from the dataset used for generating the models and reserved as controls. A total of 124 CV drugs were obtained from DrugBank, among which we could extract the features for 34 drugs, for which predictions were made. We would like to mention here that out of total 124 CV drugs, the data for targets was available only for 121. Amongst which, 94 drugs could be mapped to enzymes and 63 drugs could be mapped to transporters. While there were only 34 CV drugs which all the information i.e. targets, transporters, enzymes as well as therapeutic indications and substructure fingerprints were available. Thus the predictions were made for these 36CV drugs which had all the properties.

The most common side effects predicted were bradycardia (Disopyramide, Verapamil, Procainamide, Nifedipine, and Lidocaine); cardiac disorder (Clonidine, Telmisartan, Procainamide, and Nifedipine); congestive cardiac failure (Amlodipine and Metoprolol); cardiac murmur (Nisoldipine, Ibuprofen, and Propafenone) and tachycardia (Amlodipine, Prazosin, Bosentan, Doxazosin, Candesartan cilexetil and Acetylsalicylic acid, Telmisartan, Nifedipine and Carvedilol). These side effects had already been reported to be associated with the drugs in question on SIDER. Additionally, various side effects not reported on SIDER were also predicted by our models. The side effects include atrioventricular block (Acetylsalicylic acid, Telmisartan, Nifedipine and Midodrine); cardiomyopathy (Nifedipine [32]); cardiac failure (Verapamil [33] and Procainamide [34]); cardiopulmonary failure (Amlodipine, Prazosin, Bosentan [33], Doxazosin [33] and Bumetanide); cor pulmonale (Amlodipine, Prazosin, Bosentan [35], Doxazosin, Carvedilol, Furosemide [36] and Bepridil); and heart malformation (Disopyramide, Nisoldipine, Bosentan, Clonidine, Verapamil, Acetylsalicylic acid, Telmisartan, Procainamide, Ibuprofen, Nifedipine, Propafenone, Gemfibrozil, Metoprolol, Lidocaine, Indomethacin, Propanolol, Losartan, Felodipine, Oxepronolol, Lomitapide, Riociguat); irregular heart rate (Disopyramide [37] and Nifedipine [38]) and left ventricular failure (Bosentan, Doxazosin [39], Candesartan cilexetil, Bumetanide, Carvedilol, Furosemide, Bepridil).

Four CV drugs, Simvastatin, Benzocaine, Bezafibrate and Ezetimibe, had no side effects reported on SIDER. These drugs were predicted to be associated with heart malformation, irregular heart rate (Bezafibrate [40]), acute cardiac failure (Simvastatin [41]) and cardiac murmur (Simvastatin [41] and Bezafibrate [42]) by our classifier models.

### Predictions of uncharacterized drugs in SIDER

The predictive computational CV side effect models generated in the present study were used to make predictions on the drugs having no information of side effects on SIDER. The ADR predictions were done for twelve drugs and the most common side effects predicted included cardiac decompensation, congestive cardiac failure, cardiac disorder, cardiac murmur, irregular heart rate, shock, tachycardia and cardiopulmonary failure.

In order to make these findings significant, we performed a thorough literature search to find associations among the ADRs predicted by our models and the drugs with which the side effects were associated. Fluvoxamine was found to be related with decompensation cardiac, and fluvoxamine in combination with risperidone has been known to cause serious adverse cardiovascular drug events [43]. Various cardiac side effects were predicted for Diethylstilbestrol that include shock, congestive cardiac failure, cor pulmonale, cardiopulmonary failure, left ventricular failure, and tachycardia [44]. Mefloquine, which is an antimalarial drug was found to be associated with congestive cardiac failure and tachycardia. Anti-malarial drugs have been known to cause serious adverse cardiovascular events that include sinus bradycardia alternating with tachycardia and cardiac failure [45, 46]. Cardiac murmur, irregular heart rate, tachycardia and acute cardiac failure were the side effects observed for Famotidine which has already been reported to cause complete atrioventricular block and cardiac arrest [47]. High levels of Urea in blood have been connected to increased risk of cardiovascular events that include cardiac murmur [48], irregular heart rate [49] and acute cardiac failure [50]. Serious cardiovascular events that include block heart, cardiac murmur, irregular heart rate, cardiac failure, arrhythmia and cardiac failure acute were predicted for the drug, Eltrombopag [51–53]. According to a FDA report, Tretinoin was found to be associated with various cardiac side effects that include arrhythmias, hypotension, hypertension, cardiac failure, and cardiac murmur. Cardiac murmur, acute cardiac failure and irregular heart rate were the side effects predicted accurately by the models generated in the present study [54]. Ketoconazole in combination with Dofetilide, Pimozide, and Quinidine could also result in serious adverse cardiac effects that include tachycardia, shock, congestive cardiac failure, cardiotoxicity, cor pulmonale, heart malformation and cardiopulmonary failure [55]. Although various researchers have put forward colchicine as a probable cardiovascular agent, incidences have been reported of adverse cardiac events that include shock, tachycardia, cardiac disorder and cardiac failure [56–58]. Clomifene, Gallium nitrate and Gabapentin enacarbil were predicted to be associated with cardiac decompensation by our models; however, we could not find any literature evidence to support our observation for these drugs. Table 9 lists the CV ADRs predicted by the machine learning RF and SMO models on uncharacterized drugs in SIDER.

**Table 9:** Lists the cardiovascular ADRs predicted by the machine learning RF and SMO models on uncharacterized drugs in SIDER

Drug name	ADR predicted by RF and SMO
DB00176 Fluvoxamine	Decompensation cardiac
DB00255 Diethylstilboestrol	Shock, cardiac failure congestive, cardiopulmonary failure, left ventricular failure, cor pulmonale, tachycardia
DB00358 Mefloquine	Tachycardia, cardiac failure congestive
DB00755 Tretinoin	Block heart, cardiac murmur, heart rate irregular, cardiac failure acute
DB00882 Clomifene	Decompensation cardiac
DB00927 Famotidine	Tachycardia, cardiac murmur, heart rate irregular, cardiac failure acute
DB01026 Ketoconazole	Tachycardia, shock, cardiac failure congestive, cardiotoxicity, cor pulmonale, left ventricular failure, heart malformation, cardiopulmonary failure
DB01394 Colchicine	Tachycardia, shock, cardiac failure congestive, cardiac murmur, cardiac disorder
DB03904 Urea	Cardiac murmur, heart rate irregular, cardiac failure acute
DB05260 Gallium nitrate	Decompensation cardiac
DB06210 Eltrombopag	Block heart, cardiac murmur, heart rate irregular, cardiac failure acute, cardiac failure, arrhythmia
DB08872 Gabapentin Enacarbil	Decompensation cardiac

### Validation on external dataset

Considering the real-world application of the machine learning models generated in the present study, these models were evaluated on an external library of 16,383 MyriaScreen compound available from Sigma-Aldrich. The most common side effects predicted by RF and SVM models associated with at least 10% of compounds included cardiac murmur and decompensation cardiac. In case of RF models, cardiac murmur was predicted to be associated with 7266 compounds, tachycardia with 3346 compounds, decompensation cardiac with 3303 compounds and cardiac failure congestive with 1670 compounds. In case of SMO models, the most predicted side effect was heart rate irregular for 7272 compounds followed by cardiac murmur for 7266 compounds, left ventricular failure for 3598 compounds, decompensation cardiac with 3112 compounds, cardiopulmonary failure with 2374 compounds and cor pulmonale was associated with 2278 compounds. Few ADRs which were not predicted to be associated with any compound by any machine learning model included atrioventricular block complete, cardiac tamponade, cardiomegaly and conduction disorder. We found that the results obtained were very similar to the predictions on uncharacterized drugs.

## Discussion

The present study proposed an exhaustive computational protocol for drug ADR prediction using machine-learning based methods by integrating different levels of information for drugs. A total of 504 computational models were generated for 36 CV ADRs by integrating biological (drug transporters, targets, and enzymes), chemical (substructure fingerprints), and phenotypic (therapeutic indications and other known ADRs) features using RF and SMO machine learning algorithms. To find the informative and discriminative features, we used an mRMR approach which provided us with a list of non-redundant features of maximum relevance for classification, thereby reducing the feature space and computational time involved. Furthermore, we also employed the SMOTE method on the training data for handling data imbalance, which balanced the minority class by generating synthetic instances. The performances of the biological [accuracy 93.56% (RF) and 93.28% (SMO)] and phenotypic features [accuracy 93.83% (RF) and 93.83% (SMO)] alone were better in comparison to chemical feature [accuracy 91.41% (RF) and 88.75% (SMO)] models. The results showed that the chemical feature models were least informative in cases of both, RF and SMO models; however, the performance of the models improved upon integration of biological and phenotypic features to chemical features. To show the real-life application, efficiency and significance of the computational models, we also performed ADR prediction for uncharacterized drugs and already existing CV drugs, which were not a part of the training set used for generating the models.

## Conclusion

The present work focused on generating machine-learning based computational models for the prediction of cardiovascular ADRs. In this study, we have investigated three levels of information: biological properties including drug targets, enzymes and transporters; chemical features represented by PubChem substructures; and phenotypic properties that include drugs therapeutic indications and other known ADRs. Two machine-learning algorithms, sequential minimization optimization (SMO) and random forest (RF), were used to generate computational models trained using chemical, biological and phenotypic properties as well as their two and three level combinations for 36 CV ADRs. In conclusion, the proposed machine learning based data-integration approach could be a promising method for the prediction of potential ADRs prior to preclinical testing stages.

## Additional files

**Additional file 1.** Provides the list of the top 50 features ranked by mRMR.

**Additional file 2.** Provide the ten-fold cross-validation performance measures for the RF and SVM models for each cardiovascular ADR using biological, chemical, phenotypic features and their two and three level combinations.

**Additional file 3.** Provide the performance measures for the RF and SVM models for each cardiovascular ADR using fifty biological, chemical, phenotypic features and their two and three level combinations on non-redundant testing dataset using SMOTE data balancing method.

**Additional file 4.** Provide the performance measures for the RF and SVM models for each cardiovascular ADR using fifty biological, chemical, phenotypic features and their two and three level combinations on non-redundant testing dataset using SpreadSubsample data balancing method.

**Additional file 5.** Provide the performance measures for the RF and SVM models for each cardiovascular ADR using eighty features biological, chemical, phenotypic features and their two and three level combinations on non-redundant testing dataset.

## Data Availability

The datasets used and/or analyzed during the current study are available from the corresponding author on reasonable request.
